# Significance and popularity in music production

**DOI:** 10.1098/rsos.170433

**Published:** 2017-07-12

**Authors:** Bernardo Monechi, Pietro Gravino, Vito D. P. Servedio, Francesca Tria, Vittorio Loreto

**Affiliations:** 1 Institute for Scientific Interchange (ISI), Via Alassio 11/C, 10126 Torino, Italy; 2 Department of Physics, Sapienza University of Rome, Piazzale Aldo Moro 2, 00185 Roma, Italy; 3 Complexity Science Hub Vienna, Josefstädter Strasse 39, 1080 Vienna, Austria

**Keywords:** popular music, success, innovation, cultural evolution

## Abstract

Creative industries constantly strive for fame and popularity. Though highly desirable, popularity is not the only achievement artistic creations might ever acquire. Leaving a longstanding mark in the global production and influencing future works is an even more important achievement, usually acknowledged by experts and scholars. ‘Significant’ or ‘influential’ works are not always well known to the public or have sometimes been long forgotten by the vast majority. In this paper, we focus on the duality between what is successful and what is significant in the musical context. To this end, we consider a user-generated set of tags collected through an online music platform, whose evolving co-occurrence network mirrors the growing conceptual space underlying music production. We define a set of general metrics aiming at characterizing music albums throughout history, and their relationships with the overall musical production. We show how these metrics allow to classify albums according to their current popularity or their belonging to expert-made lists of important albums. In this way, we provide the scientific community and the public at large with quantitative tools to tell apart popular albums from culturally or aesthetically relevant artworks. The generality of the methodology presented here lends itself to be used in all those fields where innovation and creativity are in play.

## 1. Introduction

Starting from the beginning of the twentieth century popular music has acquired a great importance in modern culture. Technological progress allowed for a massive and worldwide diffusion of musical genres and styles, previously confined in relatively small contexts and milieux. This diffusion process gave birth to a global musical culture, i.e. a culture in which a large fraction of the world population shares the same perspective about styles, instruments, famous artists and so on. In this context, music-recording industries have been playing a progressively more pivotal role, with a market value in 2014 estimated at US$15 billion [1]. Music albums have been central for those industries until very recent times. Digital sharing technologies decreed the so-called ‘Death of the Album’ [2], meaning a form of music distribution now being surpassed by Internet sharing and downloading, which allow listeners to buy and enjoy one single track at a time. Despite their recent decline, albums can still be identified as the main artwork created by bands and performers since they have long represented the final product to be sold to the public. As in many other fields of artistic expression, musicians and composers aim at achieving success and popularity, i.e. being known and appreciated by a large audience. However, acquiring such a status does not necessarily imply that their albums will effectively impact the overall production in their own, or other, cultural areas. Conversely, many important albums, even those with the potential of opening up new avenues, might have experienced only a moderate recognition by the public or been long forgotten due to ageing. We shall refer to this class of albums as ‘significant’ albums and this paper will aim at identifying metrics and tools to tell under which conditions a music album can be labelled as ‘popular’, ‘significant’ or both.

In recent times, also owing to the availability of large online databases [3], many efforts have been devoted to a quantitative study of cultural and artistic systems: from the development of methods aiming at identifying new relevant creations [4,5], to the forecasting of future popularity [5–7], or the study of the evolution of the features of a particularly creative field [8–10]. A common trait of many of these works is the focus on the interrelations between the creations, modelled as nodes of a growing complex network [11]. The identification of the relevance of a work is usually inferred from its statistical properties within the network and their dynamical evolution. In this paper, we slightly change perspective and generalize this approach by analysing a different network, namely a proxy for the conceptual network where creations are embedded. To this end we focus, without loss of generality, on tags, freely chosen keywords users attach to musical albums through a social annotation process [12,13]. We consider in particular tags associated with musical albums in *Last.fm* [14], a popular music website, whose users’ contributions bootstrap a bottom-up classification, usually named ‘folksonomy’, that reflects [15] specific features of albums. Though technically a folksonomy is represented by a tripartite network, with three distinct sets of nodes, namely resources, users and tags, here we focus on a projection of it, the network of tags. Folksonomies have been shown to possess peculiar statistical properties [16], have been studied in various contexts such as photo-sharing, blogs and web bookmarking [17–19] and have been modelled mathematically within the framework of complex networks science [20,21]. In [22], it has been shown how tags in *Last.fm* define a low-dimensional semantic space at the track level, highly organized by artist and genres.

In order to investigate the impact of each new album on the future production, here we take a dynamical perspective in which every new album modifies the network of tags either by enlarging the network itself or by creating new connections. In this way, tags form a dynamical network whose size and structure is constantly reshaped through the action of users. Following the notion of the *Adjacent Possible*, as introduced by Stuart Kauffman [23] and recently formalized through a Pólya Urn process [24], we can think about this network as the growing space of musical albums’ features. The application of Pólya Urns as generative models is not new in applied mathematics and complex systems science, and transcends the application to innovation dynamics and expanding spaces. The existence of reinforcement effects in the dynamics of complex networks, for example, makes them suited to be generated with variants of Pólya Urn processes [25], featuring the ‘rich-get-richer’ effect also known as ‘Yule process’ [26]. The application to innovation dynamics requires the introduction of conditions for the arrival of innovations as formalized for the first time in the ‘Hoppe Urn model’ [27] and extended in [24] in order to model the appearance of new elements as the consequence of the introduction of novelties. In the perspective of [24], the introduction of a new album contributes not only to the growth of the network, in its year of release and through its features, but also to open up new avenues, making some novel features accessible to other users and ready to be exploited by other creations. By means of this conceptual space and its indirect relation with the set of albums, we can predict ‘ground truths’, i.e. lists of particularly relevant albums according to the criteria of either success (linked to popularity), aesthetic or historical relevance (linked to significance). In this way, we can unveil the main differences between albums belonging to different lists, as quantified though suitably defined metrics connected to success or significance. Note that a similar approach using a different tagging system has been proposed in [28], where plot-related tags were used to quantify the degree of innovation of movies. The generality of our approach makes it suitable for application in other contexts where it is important to identify the fingerprints of future popularity or long-term significance.

## 2. Material and methods

### 2.1. Dataset

Our analysis is based on a dataset, we collected by crawling the *Last.fm* website in February 2015, with the dedicated API provided by *Last.fm* itself (the data can be accessed through [29]; details of the collection of the dataset can be found in the electronic supplementary material, section A). The dataset we collected stores information about music albums, released in a time frame going from 1950 to 2015. For each album, we stored information such as the number of times an album has been listened to through the *Last.fm* platform (denoted as *Playcount* in the following), the artists, the release year, the list of songs and all user-assigned tags. Multiple releases of the same album have been excluded from the sample, keeping only the very first release. Tags are usually single words or short sentences that users may use to annotate albums to ease future retrieval. To each tag in each album is associated a score ranging from 1 to 100 indicating how frequently it has been adopted by users. For instance, a tag with a score of 100 is very significant for an album, while a score of 1 signals a less relevant tag (see the electronic supplementary material). To each album, we associate the date of its first release and we do not consider its subsequent editions. Therefore, when necessary, we correct the release date of an album by resorting to another music information archive, *MusicBrainz* (http://musicbrainz.org/) which contains the complete information on the release dates of all the editions of an album. Albums without information on their release date were filtered out of the dataset. The dataset was further filtered to correct for the presence of ‘over-tagged’ albums, namely tagged a lot by a small number of users. This results in a large number of tags with a boosted high score. As these tags are poorly validated by users, the classification of the corresponding album may be inaccurate. To solve this problem, we considered the correlations between the number of tags of an album and its playcount value, the latter being a proxy for the number of users who ever tagged the album. We used this information to identify ‘outlier albums’, which display more tags than other albums with similar playcount values, and reduced the number of their tags below a given threshold. More information about this tags-filtering process can be found in the electronic supplementary material, section A. At the end of this process, we remove from the sample all the albums without a correct release date or without tags, ending up eventually with a subsample of 163 829 time-ordered albums and 108 984 distinct tags.

### 2.2. Network of tags and topical representation

**Figure 1.**
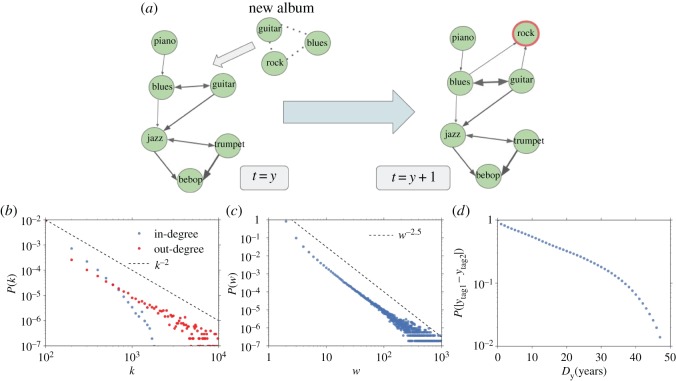
Tag network dynamics and properties. (*a*) Pictorial representation of the dynamics of the network of tags. An album is represented by a clique of tags introduced in the network at the first release date of each album. In the above example, the new album contributes both to strengthen an existing link and to add the new ‘rock’ tag to the network. (*b*–*d*) Out-degree (red) and in-degree (blue) distributions, distribution of links’ weights and distribution for the creation time differences between nodes linked in the network.

The tags associated with albums together with album release dates can be used to build a growing co-occurrence network of tags, by mutually linking tags assigned to the same album [13,30,31]. This network will be then adopted to define the properties of each album and its relation and contribution to the global music production. We start with an empty network at the first year of our albums set (1950). Every year a certain number of albums are introduced in the system according to their release dates. In this growing network G, an album is represented by a clique of tags that merges with the existing network through the addition of new tags, the creation of a new link between two already existing tags or the strengthening of a link as depicted in figure 1*a*. It is in this way, that an album dynamically modifies the network and contributes to the global music production, possibly affecting future creations. To each node of the network, i.e. each *tag*, a creation time-stamp *y*_*tag*_ is associated as the release date of the oldest album in which the tag ever appeared. In this way, the emerging network is a directed one with each link going from the eldest node to the youngest in terms of creation times. Tags created in the same year will be connected through a bidirectional link. The total number of co-occurrences of a pair of tags until a certain year *y* defines the weight of the link *e*=(*tag*_1_,*tag*_2_), *w*(*e*,*y*), where we will drop the dependence on *y* whenever the network at the final year *y*=2015 will be considered. The final (at year *y*=2015) network G exhibits a power-law distribution for the out-degrees and weights of links (figure 1*b*–*d*). On the other hand, in-degrees distribution is not as broad as the out-degrees one. Though the total number of links is large (11 801 650), about 90% of them has weight *w*(*e*)≤2. Figure 1*c* reports the distribution of the absolute difference between the creation times of the pairs of tags connected in the network. Also, here one observes an heavy-tailed distribution pointing to the existence of albums with tags created in very different times.

We exploit the tag co-occurrence network to easily cluster tags according to a common topic through a community detection algorithm. A community of nodes (cluster of tags) is a subset of nodes more strongly connected among themselves than with the rest of the network. After removing the direction of edges, we apply the Louvain method of community detection [32]. In this way, each tag is assigned with its own topic according to the community it belongs to. We assume that all the tags within the same community bear a close semantic relation. Note that when considering the growth of the network at different time frames, the community structure might vary as it is likely that many communities would not yet exist at early times, while other communities would contain elements differing from those present at the final stage. However, we will assume that the topic of a tag is defined by the latest community structure referring to the year 2015. In this way, we are somehow looking at the music production from the present-day point of view. In the electronic supplementary material, figure B, we report the fraction of the total number of tags belonging to a subset of topics every year from 1950 to 2015.

### 2.3. Album features and impact metrics

We now introduce several metrics to characterize albums based on the tags co-occurrence network and the associations topics-tags. These metrics, summarized in table 1, are meant to quantify some of the features of the albums, their mutual relationships and their impact on the overall production. We indicate a generic album as *a*, its release date as *y*_*a*_, the set of its tags as *T*_*a*_ and the set of the topics associated with its tags as *L*_*a*_. Similarly, the set of all the possible pairs of tags in *T*_*a*_, each one corresponding to an edge in the co-occurrence network, is indicated as *E*_*a*_. Given the co-occurrence network, *F*(*l*,*y*) indicates the total fraction of tags adopted for a given topic *l* until year *y*, while *T*(*y*) denotes the set of tags present in the network until the year *y*. Given the significant correlation between the playcount values and the number of tags of an album (see the electronic supplementary material, figure A), all the metrics or the quantities involved in their definition are ‘intensive’, meaning that they do not explicitly depend on the total number of tags. We will define in the following the adopted metrics, grouping them in three clusters according to the main information they carry (for a more detailed definition of these metrics, see the electronic supplementary material, section C). Our first set of metrics is related to the ‘heterogeneity’ of an album, i.e. how diversified are the tags describing it. It has been shown in other contexts [33] that being heterogeneous can be an advantage in terms of acquiring popularity, because the more diversified the content, the larger is the variety of public who might be interested.

**Table 1.** Metrics characterizing music albums and their relationships with the tag co-occurrence network.

name	formula	main quantities/notation
topical entropy	$E(a)=\frac{-\sum_{l\in L_{a}}f(l)\log f(l)}{\log|L_{a}|}$	*f*(*l*)=*fraction* of tags in *T*_*a*_ with label (topic) *l*
average time span	$TS(a)=\frac{\sum_{e\in E_{a}}|y_{{\mathrm{tag}}_{2}}-y_{{\mathrm{tag}}_{1}}|}{|E_{a}|}$	*y*_*tag*_=*creation* time of the tag *tag*
average tag age	$A(a)=\frac{\sum_{\mathrm{tag}\in T_{a}}(y_{a}-y_{\mathrm{tag}})/(y_{a}-1950)}{|T_{a}|}$	
mainstreamness	$M(a)=\frac{1}{\sqrt{2}}∥\sqrt{f}-\sqrt{F}{∥}_{2}$	$\frac{1}{\sqrt{2}}∥\sqrt{.}-\sqrt{.}{∥}_{2}=\mathrm{Hellinger}$ distance
burstiness	$B(a)=\sum_{l\in L_{a}}f(l)\frac{N_{l}(y_{a}+1)-N_{l}(y_{a})}{N_{l}(y_{a})}$	*N*_*l*_(*y*_*a*_)=*number* of tags with label *l* until year *y*
novelty	$f_{\mathrm{new}}(a)=\frac{|T_{a}\setminus T(y_{a}-1)|}{|T_{a}|}$	|.∖.|= set difference
adjacent possible	$Adj(a)=\frac{\sum_{\mathrm{tag}\in T_{a}}f_{\mathrm{Adj}}(\mathrm{tag},y_{a})}{|T_{a}|}$	*f*_*Adj*_(*tag*,*y*_*a*_)=*fractions* of nodes entering in the adjacent possible of *tag* during *y*_*a*_.
Uchronia	$uch(a)=\frac{1}{|T_{a}|}\sum_{\mathrm{tag}\in T_{a}}\text{uch}(\text{tag})\;\left(1-\frac{N(\mathrm{tag},y_{a})}{N(\mathrm{tag})}\right)$	*uch*(*tag*)=*percentage* of G destroyed by the removal of *tag*
		*N*(*tag*)=*number* of albums containing *tag*
		*N*(*tag*,*y*_*a*_)=*number* of albums containing *tag* till year *y*_*a*_
Uchronia entropy	$h_{uch}(a)=\frac{1}{|T_{a}|}\sum_{\mathrm{tag}\in T_{a}}h_{uch}(\mathrm{tag})\;\left(1-\frac{N(\mathrm{tag},y_{a})}{N(\mathrm{tag})}\right)$	*h*_*uch*_(*tag*)=*variation* of the entropy of *F* after the removal of *tag* from G.

*Topical entropy* *E*(*a*) is the entropy of the distribution of the tags *T*_*a*_ associated with the album *a* over the set of topics *L*_*a*_, normalized in [0,1]. A value close to 1 denotes an album less biased on a single topic. If topics represent musical styles, the album with an entropy close to 1 can be considered as a balanced mixture of many styles. Heterogeneity can also be relevant in time as witnessed by the large distribution of creation times as seen in figure 1*d*. For instance, *classicrock* and *synthrock* tags belong to the same topic but they have been introduced in very different times. *Average time span* *TS*(*a*) quantifies this kind of heterogeneity measuring the average creation time distances between the set of tags of an album, so that this metric is 0 if all the tags have been created in the same year. The *average tag age* *A*(*a*) is defined as the average relative age of all the tags associated with the album *a*, and it aims at quantifying the interplay between age of a tag and its impact or success.

The following set of metrics quantify the relationship of an album with the music production preceding it. The *mainstreamness* *M*(*a*) measures how different was the album with respect to the musical production before its release and it is defined as the distance between the probability distribution of the tags associated with the album and the global probability distribution of all tags associated with all the preceding albums. A value of *M* close to 0 indicates an album fully aligned with the previous production, while a value of 1 denotes strong originality.

*Burstiness* *B*(*a*) identifies whether an album has contributed to a significant increase in the production associated with a given topic. It is defined as the weighted average of the logarithmic derivative of the number of tags belonging to each topic in *L*_*a*_ in the release year *y*_*a*_ of an album (see the electronic supplementary material, figure C for a representation of the logarithmic derivative of the number of tags in some topics). A large value of *B*(*a*) means that the album was released in a year when one or many of its topics have experienced a fast relative growth, indicating that the album was created following an emerging trend. Finally, the *novelty* metric *f*_new_(*a*) is the fraction of tags in *T*_*a*_ which are actually new at the time *y*_*a*_, which is a proxy for the innovations introduced by the album *a*.

**Figure 2.**
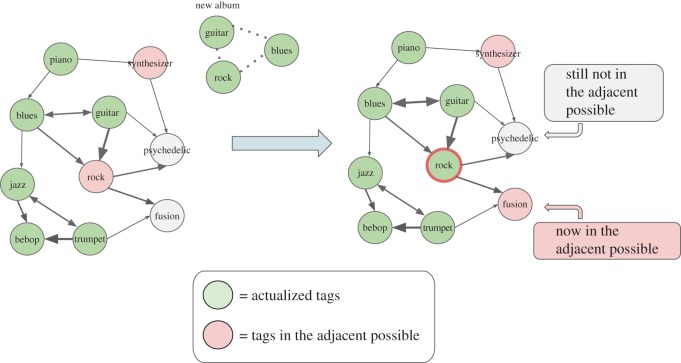
Adjacent possible. Cartoon illustrating the structure and growth of the adjacent possible space after the release of a new album labelled with the three tags *guitar*, *rock* and *blues*.

Another interesting feature to look at concerns the notion of ‘possible’. Instead of looking only at the relationship of an album with its preceding production, one can focus on the new possibilities opened by the introduction of an album and its long-term impact on the network. Following Kauffman [23], the *adjacent possible* of an album *a* can be defined as the set of all tags unlocked, i.e. made possible, by the release of the album and of its associated tags. To be more precise, we imagine first the complete co-occurrence graph of tags as seen by 2015. This includes all the tags (and their links) ever adopted by the albums released during the whole time span considered. At the beginning of the evolution, this graph has not been explored yet and all the tags are grey, as in figure 2.

As time goes by and new albums are released, tags are progressively coloured in green, meaning tags actually adopted for released albums (we shall refer to them as *actualized*). A third category of tags can also be identified at each time step, namely tags belonging to the adjacent possible space of a given actualized tag, with the following definition. Given an album *a* released at time *y*_*a*_ and its set of tags *T*_*a*_, a tag belongs to the adjacent possible space of *a* if it belongs to the adjacent possible of any given tag *tag*∈*T*_*a*_. The adjacent possible space of a tag *tag*∈*T*_*a*_ is composed by all the tags that: (i) are neighbours of *tag* in the global co-occurrence graph; (ii) have been not yet adopted by users at time *y*_*a*_, and (iii) for which all its predecessors (i.e. all the tags with in-links to them) have all been already adopted by users. With reference to figure 2, the tag *Fusion* enters in the adjacent possible space of *rock* because all its predecessors (*trumpet* and *rock*) have been already actualized. In other words, the adjacent possible space of an album quantifies the number of tags that are one step away from being introduced after its release. In more detail, we define *Adj*(*a*) of an album *a* as the average, over *tag*∈*T*_*a*_, of the fraction of nodes entering the adjacent possible space of *tag* during *y*_*a*_. This definition makes it easy to identify which part of the co-occurrence network is ready to be discovered by future music production, because all the ingredients necessary for its existence are already in place. The introduction of each new tag contributes to the expansion of this space of possibilities, because such a tag might be the last needed predecessor for other tags to become part of the adjacent possible space. A value of *Adj*(*a*) close to 1 indicates that almost every neighbouring tag of *T*_*a*_ potentially triggered by the release of *a* is now very close to being adopted. On the other hand, a value of *Adj*(*a*) close to zero indicates that either the album features a set of tags lying in a part of the network already widely explored or that the album is too ‘avant garde’, because all the nearby part of the co-occurrence network cannot be adopted as the predecessors are not yet in place. In summary, the adjacent possible metric quantifies the short-term impact of an album, identifying how much of the potential adjacent possibilities are actually unlocked by the album itself. A metric similar to the one used here has already been introduced in [34] in the study of the cultural evolution of the production of movies, pointing out its relation with the cultural value of movies.

To quantify the long-term impact of an album on the subsequent production, we resort to an approach inspired to network resilience theory [35,36]. Given a certain *tag*∈*T*_*a*_, we remove such a tag from the network and, following its out-links, we iterate this removal process for its neighbouring tags until no further removal is possible. Links connecting removed tags are also removed, so that one ends up with a smaller network $G^{\prime }$. We now define *uch*(*tag*) as the fraction of G destroyed by the removal of *tag*. *Uchronia* *uch*(*a*) is obtained by averaging *uch*(*tag*) over all *tag*∈*T*_*a*_ (weighted to account for how many new albums adopted *tag* after the release of album *a*) and it quantifies the impact of an album on future production.

Connected to *Uchronia* is *Uchronia entropy*, *h*_*uch*_(*a*), i.e. the variation of the entropy of the distribution of tags over the different topics, averaged over all *tag*∈*T*_*a*_. *Uchronia entropy* quantifies how much the removal of the tags of an album impact the topical distribution of tags. For example, a negative value of this quantity indicates that the removal has brought us to a more uniform distribution, destroying tags clustered around one or more larger topics. On the other hand, a positive value of *h*_*uch*_(*a*) indicates that the final distribution is more clustered, so that just one or few more topics gather all the remaining tags.

## 3. Results

### 3.1. Metrics correlations and comparison with standard network metrics

**Figure 3.**
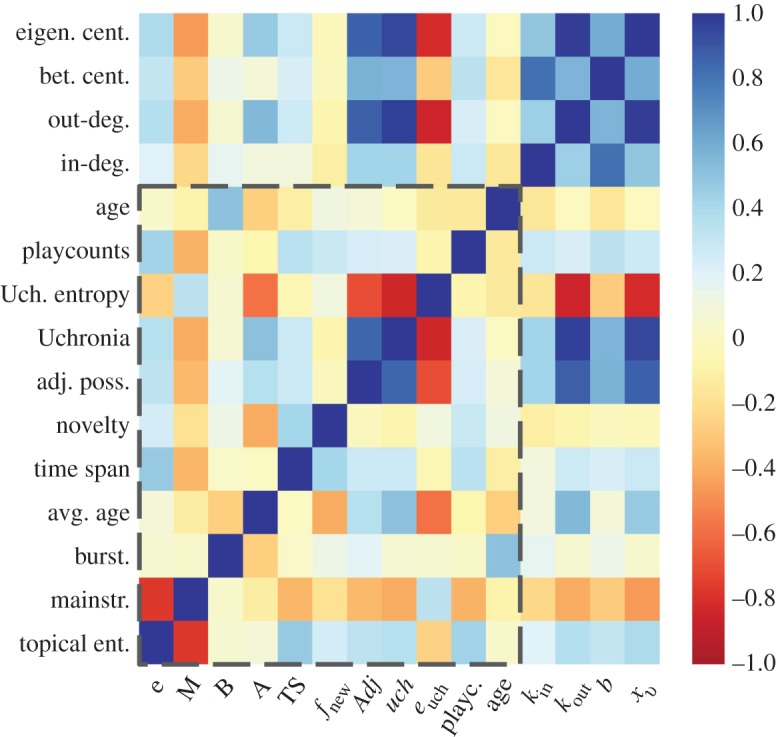
Spearman’s correlation coefficients between all the metrics introduced in table 1, plus playcount values and album age within the dotted square. Metrics outside the dotted square are standard network centrality metrics (in-degree, out-degree, betweenness centrality and eigenvector centrality). All the positive and negative coefficients were found to be significant, with a *p*-value considerably smaller than 0.05.

Figure 3 shows the correlation matrix between all the metrics introduced above, the playcount values of each album and their age in 2015 (rows and columns within the dotted square). There are clear strong anti-correlations between the mainstreamness and the topical entropy of an album and between its two long-term impact metrics, *Uchronia* and *Uchronia entropy*. The interpretation of the latter case is quite trivial: destroying a large fraction of the network leads to a more entropic situation, where larger topics have been considerably reduced and the ensuing distribution is more uniform. The anti-correlation of the *mainstreamness* and the *topical entropy* expresses that small topical diversities are usually related to some specialized ‘niche’ albums, far from the mainstream production. Playcount values exhibit slight correlations (Spearman coefficients all significant but never larger than 0.36 in absolute values) with almost every metric except *burstiness* and *average tag age*. The small anti-correlation between playcount values and age (Spearman coefficient −0.14) indicates that being old does not imply a strong influence on the current success of an album, and this is probably due to the recommendation system of *Last.fm*, favouring novel albums. The relationship between all the metrics and playcount values are better shown in section C of the electronic supplementary material, file S1. The metrics introduced have also been compared with well-known network centrality metrics such as in-degree, out-degree, betweenness centrality and eigenvector centrality, corresponding to the rows and columns outside the dotted square in figure 3. These metrics are computed over the co-occurrence network whose nodes are tags, and we assigned to each album the value corresponding to the average over its tags. We can see that these metrics are highly correlated with each other, and feature the same correlation patterns with the other metrics introduced in this work, i.e. they are correlated or anti-correlated in the same way with the other metrics. Hence, they seem to carry similar information regarding the properties of the albums. Unsurprisingly, they exhibit larger (anti-)correlations with the Uchronia, Uchronia entropy and adjacent possible metrics, which can be regarded as network centrality metrics.

### 3.2. Popularity and significance

The popularity of a given album is strongly linked to its commercial success, connected, in its turn, to the number of people who have listened to it since its release date. Playcount values on *Last.fm* represent a good proxy to assess popularity. Through them we define as ‘popular’ albums with playcount values larger than the 75th percentile of the distribution (≈10^5^) and as ‘highly popular’ albums with playcount values larger than the 90th percentile of the distribution (≈10^6^). As already mentioned, popularity does not always coincide with significance. In order to go beyond popularity and quantify the level of significance of an album, we make use of expert-made lists of influential albums. In this way, we rely on experts’ judgements about what is ‘significant’ or ‘worth’ and we can investigate whether those albums feature important differences with respect to albums in the popular and highly popular classes. We considered in particular the following lists, easily accessible from the Internet:

— *The 500 Greatest Albums of All Time according to the Rolling Stone Magazine* (RSM; http://www.rollingstone.com): a list of the 500 most important albums according to the votes expressed by selected rock musicians, critics and industry persons. A total of 387 out of 500 albums were also present in our dataset.
— *The 500 Greatest Albums of All Time according to the NME Magazine* (http://www.nme.com): similar to the previous one, it is based on the votes from current and past NME journalists. Here, 376 out of 500 albums were also present in our dataset.
— *Grammy Hall of Fame* (GHF; https://www.grammy.org): a hall of fame to honour recordings of lasting qualitative or historical significance. Every record from any musical genre is eligible to be part of the list as long as they are more than 25 years old. We identified 322 entries in our dataset being also part of the GHF.
— *The National Recording Registry (*NRR*)*: a list of recordings that are considered culturally, historically, or aesthetically important or relevant to the American Culture (https://www.loc.gov/programs/national-recording-preservation-board/recording-registry/). We identified 230 entries in our dataset being also part of the NRR list.

Table 2 shows a brief list of the five albums with largest playcount values for every list. Despite the presence of some overlaps, the lists are quite different from one another and reflect different viewpoints on music production.

**Table 2.** First five most popular album in the 4 expert-made lists. Playcount of *Last.fm* and release date are also shown.

rank	RSM	playcount	release year
1	Radiohead: In Rainbows	65 015 418	2006
2	Arctic Monkeys: Whatever People Say I Am, That’s What I’m Not	59 064 721	2005
3	Radiohead: OK Computer	55 725 336	1996
4	MGMT: Oracular Spectacular	53 474 495	2006
5	Coldplay: A Rush of Blood to the Head	49 335 385	2001

rank	NME magazine	playcount	release year
1	Radiohead: In Rainbows	65 015 418	2006
2	Radiohead: OK Computer	55 725 336	1996
3	MGMT: Oracular Spectacular	53 474 495	2006
4	The Killers: Hot Fuss	52 243 385	2003
5	Coldplay: A Rush of Blood to the Head	49 335 385	2001

rank	GHF	playcount	release year
1	The Beatles: Abbey Road	38 558 915	1968
2	The Beatles: Revolver	23 217 206	1965
3	The Beatles: Sgt. Pepper’s Lonely Hearts Club Band	22 499 864	1966
4	The Clash: London Calling	21 916 273	1978
5	Pink Floyd: The Wall	19 662 592	1978

rank	NRR	playcount	release year
1	Radiohead: OK Computer	55 725 336	1996
2	Nirvana: Nevermind	38 299 519	1990
3	The Beatles: Sgt. Pepper’s Lonely Hearts Club Band	22 499 864	1966
4	Pink Floyd: The Dark Side of the Moon	14 292 759	1972
5	The Doors: The Doors	14 226 647	1966

**Figure 4.**
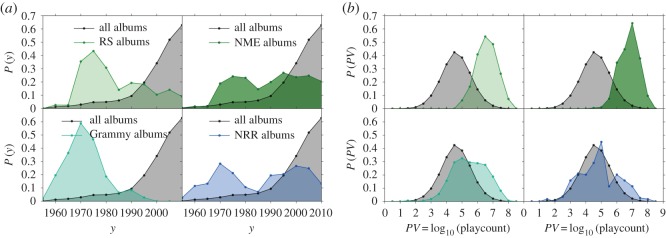
Playcount and release date distributions. Comparison between the distribution of the release dates (*a*) and the playcount values (*b*) for all the albums in our datasets and those included in the four expert-made lists described in the text.

Figure 4 shows the distribution of the release dates and the playcount values of the albums for the four lists compared with the whole sample. Both the RS and the GHF lists have a bias towards albums produced between the 1960s and the 1980s, while the other two lists seem to be less biased in this sense and more uniform in time. On the other hand, the only list that seems not to be biased towards popular albums is the NRR list, followed by the GHF list showing just a weak bias. Hence, we decided to merge the RS and NME lists and the GHF and NRR lists. In this way, we obtained two larger lists indicated in the following with the names ‘RS/NME’ and ‘GHF/NRR’, the first one being more biased towards popularity than the second. These two categories will be then compared with the popular and highly popular ones. Summarizing, we will consider the following overlapping categories:

— *popular category*: 41 053 albums with a playcount value larger than 10^5^.
— *high-popular category*: 7325 albums with a playcount value larger than 10^6^.
— *RS/NME category*: 619 albums considered important by the RSM and NME magazine.
— *GHF/NRR category*: 509 albums listed as within the GHF and the NRR.

**Figure 5.**
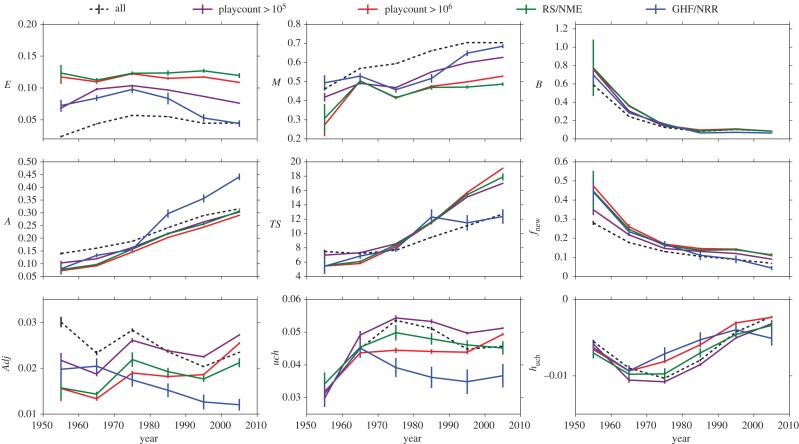
Time behaviour of album metrics. Average value of the album metrics as a function of their release dates. Black dashed lines are the averages over the whole sample of albums, while different colours correspond to different category of albums.

Figure 5 shows the behaviour of the metrics defined in the previous section as a function of time for these categories. The qualitative behaviour of the metrics restricted to one of the classes is usually quite similar to that of the whole sample of albums. However, it is evident that they usually display a higher degree of heterogeneity (*topical entropy* and *average time span* are higher with respect to the whole sample) and have a small mainstreamness. In the electronic supplementary material, section F, we also show the playcount values as a function of our metrics for the four defined categories. Again we find qualitatively similar behaviours to the whole sample in every case, with very few exceptions. Finally, figure 5 shows the behaviour of these metrics in time. For many metrics the trends are trivial. For instance, the *burstiness* is higher for older albums meaning that at early stages it is somehow easier to trigger a great relative growth of a given topic; the *average tag age* *A*(*a*) and *average time span* *TS*(*a*) increase in time owing to the availability of older and older tags; novelty *f*_new_(*a*) decreases due to the difficulty of introducing new elements after a lot of them are already present (similar to what happens with Heap’s Law for texts and other systems [24,37]). Other metrics instead do not display clear monotonic trends. For instance, the adjacent possible metric related to the very next future stays more or less constant in time. Long-term impact metrics (*uch*(*a*) and *h*_*uch*_(*a*)) and the topical entropy *E*(*a*) feature instead a positive or negative peak around 1970 and 1980, indicating a time frame of great heterogeneity in which many influential tags have been introduced.

#### 3.2.1. Predicting album category

From the plots in figure 5, it is not easy to assess whether the expert classifications are somehow different from the overall sample and what are the differences among one and another. In order to see whether our metrics are predictive either of success or of the belonging to the RS/NME or GHF/NRR categories, we performed a random forest classification [38] using the album metrics defined in previous sections as predictors. For each classification, we use a training set corresponding to the 80% of our data points, chosen at random without replacement. In order to have a good and unbiased classification, we ran the algorithm by using 1000 random trees, weighting each class according to their size in order to compensate for the high unbalance between them. After having trained the algorithm, we built a receiver-operating characteristic (ROC) curve based on false and true positives rates in the remaining sample. The Area Under the ROC curve (AuROC) is a quantification of how good our classifier is. An AuROC equal to 0.5 means that our classifier is basically random, while 1 indicates a perfect classification.

In every case, we find that our predictors are generally able to classify quite correctly the albums: we have an AuROC equal to 0.85 and 0.92 for the classification of albums with playcount values larger than 10^5^ and 10^6^, respectively; an AuROC equal to 0.89 for the RS/NME list; AuROC equal to 0.81 for the GHF/NRR list. In order to test the significance of the predictions, in the electronic supplementary material, section D, we show the same prediction for a randomized case where both belonging to a category and the playcount have been randomly reassigned among all the albums being released in the same decade. The classification for this randomized case shows a significant decrease of the AuROC for each category, being in most cases very close to 0.5.

The random forest algorithm (RFA) provides also information about which metrics are more relevant for the prediction. The RFA delivers a score by which the examined predictors can be ranked. These scores take into account the differences between the prediction errors obtained with the actual data, and a randomly reshuffled dataset [38]. In this way, we have information on what metrics have been mainly used to predict the belonging of an album to one of our categories and to eventually identify differences.

**Figure 6.**
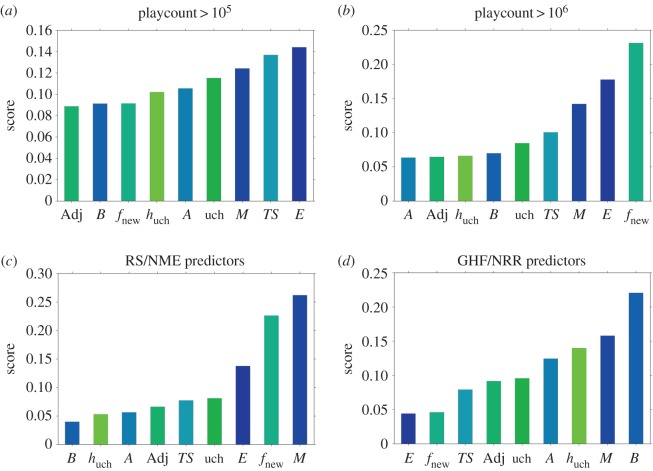
Album metrics ranking according to the random forest algorithm. Ranked scores of the metrics used for the prediction of the four categories: (*a*) popular, (*b*) highly popular, (*c*) RS/NME and (*d*) GHF/NRR.

In figure 6, we show the ranking of the metrics used for the prediction of our categories. Most of the metrics introduced in this work depend on the particular community structure we considered. In all the results discussed above, we used the Louvain method for community detection [32]. In order to test the robustness of our predictions with respect to variations in identifying communities, we repeated all the measures when the community structure is identified by using the OSLOM algorithm [39]. In the electronic supplementary material, section E, we show that even with this different method we are able to predict belonging to each category with almost the same accuracy. Moreover, the most important metrics for the various predictions remain the same.

#### 3.2.2. Popular category

**Figure 7.**
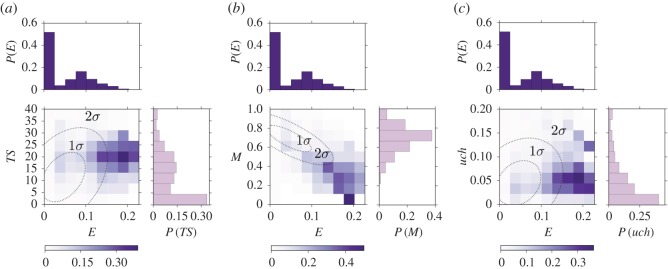
Top-ranked metrics for the category *popular*. Measured probability of being in the category *popular* as a function of pairs of the four highly ranked metrics used for the prediction with the RFA. Darker colour indicates bins with larger probability. The two dotted dashed lines are the ellipses of covariance of the two considered metrics with 1 and 2 standard deviations. On the side of the axes the distributions of the metrics are also shown. The couples of metrics used are (*a*) topical entropy–average time span, (*b*) topical entropy–mainstreamness and (*c*) topical entropy–Uchronia.

The most important features for the *popular* category classification are the heterogeneity metrics (*E*(*a*), *TS*(*a*) and *A*(*a*)), followed by mainstreamness *M*(*a*) and Uchronia *uch*(*a*). Note that the *popular category* being the largest (25% of the sample can be considered as part of this category), it is also the one which is most difficult to classify as it contains albums with quite diverse features. In fact, the first three metrics in the ranking account for less than $\frac{2}{5}$ of the total score and the score of every feature is comparable. Figure 7 shows the percentage of albums belonging to the *popular* category as a function of three pairs of the most relevant metrics: topical entropy *E*–average time span *TS*, topical entropy *E*–mainstreamness *M* and topical entropy *E*–Uchronia *uch*. In other words, we show the probability that an album belongs to the category *popular* conditioned on having a certain value for a couple of these three metrics. The distributions of these metrics and the ellipses of covariance at 1 and 2 standard deviations of the scatter plot of the metrics are also shown in order to highlight where the majority of the sample is clustered. The picture emerging from crossing topical entropy and average time span (figure 7*a*) is what is to be expected from the relationship between heterogeneity and success. The probability that an album belongs to the *popular* category is enhanced by having both these metrics higher than usual and the peak of the probability lying outside the largest ellipse of covariance.

Figure 7*b* unveils another property, which will be common also for the other categories: the probability of belonging to the *popular* category is enhanced for small values of mainstreamness (indicating great similarity to the musical production at the release time of the album), as can be seen from the fact that there is a peak of probability in the bottom right corner of this panel. This is not surprising at least for the prediction of popularity (we also refer to the electronic supplementary material, figure G), as too original albums might be perceived as weird and then not acquire public favour. Finally, the comparison with Uchronia reveals that the probability of belonging to the *popular* category is higher when this value is just slightly larger than 0, indicating that albums in this category usually have an impact to some extent on the music production.

#### 3.2.3. Highly popular category

**Figure 8.**
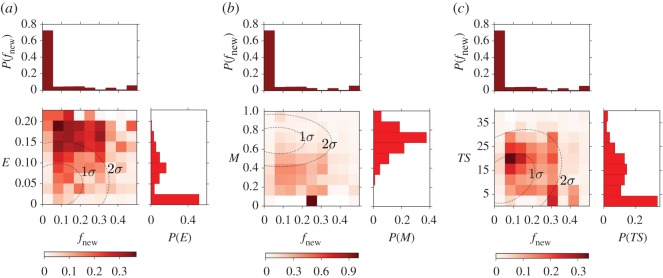
Top-ranked metrics for the highly popular category. Measured probability of being in the *highly popular* category as a function of couples of the four highly ranked metrics used for the prediction with the RFA. Darker colour indicates bins with larger probability. The two dotted dashed lines are the ellipses of covariance of the two considered metrics with 1 and 2 standard deviations. On the side of the axes the distributions of the metrics are also shown. The couples of metrics used are (*a*) novelty–topical entropy, (*b*) novelty–mainstreamness and (*c*) novelty–average time span.

For this category, two of the most relevant metrics are again related to heterogeneity, but the main predictor in this case is novelty, which roughly measures the degree of innovation of an album. Mainstreamness is again important, which is not surprising for the same reasons explained for the popular category. Note that, unlike the *popular* category, now the first three metrics account for more than 50% of the total score. Figure 8*a* shows the probability of belonging to this category as a function of the novelty and topical entropy metrics, similarly to what has been shown in figure 7 for the *popular* category. We can see that the vast majority of albums have a value of novelty which is equal to 0 (top histogram in this panel), but from the plot we can see that albums with a value larger than 0 are more likely to be part of the *highly popular* category. Moreover, we can observe in figure 8*a*,*b* that similar to the *popular* category, *highly popular* category albums have small values of mainstreamness and large values of topical entropy. Hence, with respect to the previous prediction, now heterogeneity and adherence to the style of the year of release of the album make the album more likely to be popular. However, in order to acquire vast popularity, an album should also introduce some innovations to the music scenario, suggesting that the public rewards innovative elements with most attention.

#### 3.2.4. Rolling Stone/NME category

We have seen in figure 8 that this category is strongly related to popularity. In fact the main predictors are almost the same as for the *highly popular* category even though they are ranked in a slightly different way. However, mainstreamness and novelty seem to share a larger part of the total score. The fourth relevant metric that has changed with respect to the *highly popular* case is Uchronia, a long-term impact metric, though with a very small score.

**Figure 9.**
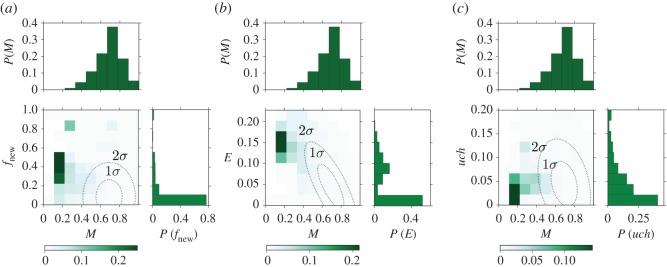
Top-ranked metrics for the RS/NME category. Measured probability of being in the RS/NME category as a function of pairs of the four highly ranked metrics used for the prediction with the RFA. Darker colour indicates bins with larger probability. The two dotted dashed lines are the ellipses of covariance of the two considered metrics with 1 and 2 standard deviations. On the side of the axes the distributions of the metrics are also shown. The couples of metrics used are (*a*) mainstreamness–novelty, (*b*) mainstreamness–topical entropy and (*c*) mainstreamness–Uchronia.

In fact, in figure 9 we can see that mainstream albums (small *M*) introducing a certain number of new tags (relatively high *f*_new_) are more likely to be part of this category. A large topical entropy also enhances the probability (figure 9*b*), while in the case of Uchronia (figure 9*c*) we can see that the probability is enhanced when the value of this metric is slightly larger than 0, indicating the need for some sort of long-term impact in order to be considered important by music magazines. The similarity between the *highly popular* albums and those belonging to the RS/NME category is quite interesting considering that the RS/NME lists are expert-made. This fact might be the effect of the mutual influence loop between magazines and public opinion. From one side, magazines are biased towards what the vast public likes in order to maximize their sales, while at the same time the public is affected by influential magazines in their opinion of what is interesting in the music scene.

#### 3.2.5. Grammy Hall of Fame/National Recording Registry category

**Figure 10.**
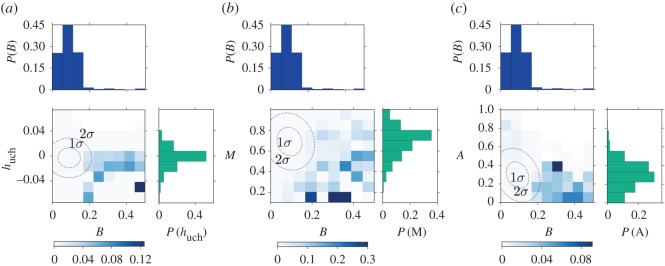
Top-ranked metrics for the GHF/NRR category. Measured probability of being in the GHF/NRR category as a function of pairs of the four highly ranked metrics used for the prediction with the RFA. Darker colour indicates bins with larger probability. The two dotted dashed lines are the ellipses of covariance of the two considered metrics with 1 and 2 standard deviations. On the side of the axes the distributions of the metrics are also shown. The pairs of metrics used are (*a*) burstiness–mainstreamness, (*b*) burstiness–Uchronia entropy and (*c*) burstiness–average tag time.

The last category, obtained by merging Grammy Hall of Fame and NRR lists, is instead clearly not related to success and popularity. Besides mainstreamness, which is the second most important predictor, the other most important are in fact quite peculiar with respect to the other categories: burstiness, Uchronia entropy and average tag age. Considering burstiness and mainstreamness in figure 10, it is evident that small mainstreamness and large burstiness are two main features of the albums belonging to this category. The picture is quite surprising when burstiness and average tag age are crossed together (figure 10*c*). While it is clear that burstiness dominates the prediction as there are large conditional probabilities for large value of burstiness, average tag age must instead be kept small. Finally, the comparison with Uchronia entropy indicates that this long-term impact metric is important, so that the probability of belonging to the GHF/NRR category is higher when this metric is negative.

The criterion of belonging to this category is drastically different with respect to the other categories we have seen and definitely not related to the taste of the public. The fact that burstiness is the main predictor indicates that the albums present in the GHF/NRR lists are typically released whenever some topic was rapidly growing, so in other words these albums could have contributed to the emergence of some trend in music production. The small values of the average tag age instead indicate that the features of the albums were highly related to the particular historical period in which they were released. Finally, the negative values of Uchronia entropy indicate that such tags have a strong influence on the future albums. The result from mainstreamness again indicates, however, that though innovative, these albums are not distant from the musical trends of their times, indicating that also in terms of influence over musical production being too far with respect to what is generally appreciated might lead to negative results.

As a final remark, we must note that the adjacent possible metric was not among the most relevant ones in every prediction. This could either be due to a real irrelevance of this metric or to a correlation with other metrics which are effectively more important. In the electronic supplementary material, section G, we show the same plots shown before for the four categories in the case of the adjacent possible metric. These plots do not highlight a particular relevance of the adjacent possible space in any of these predictions. To shed some more light on the possible meaning of this metric, we show in the electronic supplementary material, figure I, that the probability of belonging to one of the categories considered above, conditioned on having a certain value of the adjacent possible metric, features a maximum before decreasing for large values of the metric itself. It seems thus that there exists an optimal stadium of exploration of the adjacent possible that is rewarded by every category. Too avant garde albums instead, which unlock a large part of the conceptual space, are not considered valuable either by the public or by music experts.

## 4. Conclusion

Popular music is a highly competitive world, where bands and musicians produce music albums and tracks rivalling each other in order to acquire public attention. Popularity and in general commercial success seem to be the driving forces of the system, so that the production of artworks has to cater to the public’s taste. However, there are other possible levels of importance that only experts in the particular field might be able to recognize. In order to assess and characterize the presence of this new kind of significance, we developed some general metrics aimed at describing the features of musical albums, their relationships with each other and with the global music production and their impact on it. These metrics have been developed using a dynamical space of album features represented by user-defined tags collected on *Last.fm* in 2015. This space can be represented as a growing network of co-occurrence of tags, where albums are described by cliques of tags attached to the network in their first year of release. The metrics we defined were then used to predict the belonging of an album to four different categories, two of which were linked with popularity and two built using expert-made lists of important albums. Our metrics have been used as features in a random forest classification algorithm, showing a great degree of prediction accuracy (values of AuROC larger than 0.8 in every case). Moreover, the RFA gave us information on which metrics were more important for the predictions, allowing us to have some insights on how an album belonging to a category typically is. We have seen that popularity is linked with heterogeneity, so a popular album connects different musical features which might also belong to a different period of time. Another important characteristic, relevant also for the two expert-made categories, is the mainstreamness, i.e. being somehow aligned with the general style of its own period. In general, albums which could have been found ‘too weird’ did not acquire popularity.

Albums belonging to the first expert-made category, built using the albums list from the RS and NME magazines, showed a great similarity with highly popular albums. In this sense, it is possible to argue that the level of significance considered in those lists is indeed success, which is not too surprising considering that music magazines have also to cater to the general taste. Finally, albums in the last considered category, obtained by merging lists compiled by the GHF and the NRR, are instead quite different from those having acquired success and can be considered as excelling in significance. This category takes into account cultural, historical and aesthetic importance, and albums belonging to it usually have a high impact on the future evolution of the tags co-occurrence network. Most importantly, these albums were released in a period of fast growth of the musical area they contributed to. In this sense, such albums might have been seminal for important future trends and the high impact of their tags indicates that they had a strong influence on their own field. It is important to mention that a large fraction of albums in this category cannot be considered popular, so that their significance cannot be related to success.

We think the approach proposed in this paper to tell apart significance and popularity is sufficiently general to be applied to other realms of artistic expression along with all the areas of scientific production. The evaluation of the scientific impact of articles and the importance of a scholar, for instance, is nowadays represented by the h-index, computed over the distribution of the scientific citations of a given element [40], which has been widely criticized due to its biases and its lack of accuracy [41,42]. Interesting future developments of this work might include the investigation of solid criteria to decree the value of artworks and scientific productions besides success and historical relevance, which could lead to new ways for their evaluation.

## Supplementary Material

Supporting Information

## Data accessibility

All the data supporting our findings can be downloaded from http://dx.doi.org/10.5061/dryad.jc8kd [43].
