# Supplementary material for: Significance and popularity in music production
Source: R Soc Open Sci. 2017 Jul 12;4(7):170433. doi: 10.1098/rsos.170433 (PMC5541564; doi:10.1098/rsos.170433)
Supplement: Supporting Information [file rsos170433supp1.pdf]

Significance and Popularity in Music Production

Supporting Information File

Bernardo Monechi[1], Pietro Gravino[2], Vito D. P. Servedio[3], Francesca Tria[2,1,3], Vittorio Loreto[2,1,3]

1 Institute for Scientific Interchange (ISI), Via Alassio 11/C, 10126 Torino, Italy
2 Physics Dept., Sapienza University of Rome, Piazzale Aldo Moro 2, 00185 Roma, Italy
3 Complexity Science Hub Vienna, Josefstädter Strasse 39, 1080 Vienna, Austria

A Dataset Download and Tag Filtering

The *Last.fm* music platform[1] is highly connected with another online database called *Music Brainz*[2], being the same elements (songs, albums, artists) marked with the same identifier. By resorting to the *Last.fm* API [3] we downloaded a list of about 500,000 albums present both in *Last.fm* and *Music Brainz* databases. Then, we downloaded all the meta-data related to those albums such as *release date*, *track list*, *tags* and *similar albums*. Since the release date of albums in *Last.fm* might not coincide with the date of their first release, but rather with the date of the release from which the tracks where taken, we used *Music Brainz* to download all years of every release of the albums and assigned the least recent to each album in the database.

Tags in *Last.fm* are user-defined and user-validated, in the sense that a user can assign whatever tag he/she may like to an album and other users can validate it. In fact, each tag, say a, in a given album has a score ranging form 1 to 100, measuring the percentage of users that assigned or voted a tag in that album and voted a. Since there is no limit on the number of tags that a user can assign to a given album and the information about how many users have effectively voted is not available, it is possible to observe a sort of "over-tagging" from a small set of users. In other words, it may happen that very few users assigned a lot of tags to less popular albums, and each tag will result to have a high score since there were few voters. To solve this issue, we used the "Playcount" information (i.e., the number of time an album has been listened) as an upper bound for the number of tagging users . Fig A shows the scatter-plot between the Playcount of an album and its number of tags. There is a clear non-linear correlation between these two quantities (Spearman's correlation coefficient 0.54 with p-value $\ll 10^{-6}$), especially for albums with a large number of Playcounts. We performed a binning of the logarithm of the Playcounts and for each bin we examined the distribution of the number of tags. We determined the 95th percentile of this distribution, shown in Fig. A as a continuous red line, and chose it as a threshold over which an album is considered over-tagged. For each over-tagged album, corresponding to all the point above the red line, we ranked its tags in decreasing order according to the score provided by *Last.fm*. Then, we purged all the tags below a certain rank so that the final number of tags lay within the value defined by the 95th percentile of the tag number distribution in the corresponding Playcount bin. Moreover, we also manually excluded tags related to the personal preferences of *Last.fm* users (e.g., tags containing the words "favorite", "best", "iown" were removed), removing from the sample all the albums without tags at the end of this process.
After the whole filtering process, we ended up with a sub-sample of 163,829 time-ordered albums and 108,984 distinct tags.

[1] www.lastfm.com
[2] musicbrainz.org
[3] http://www.last.fm/en/api

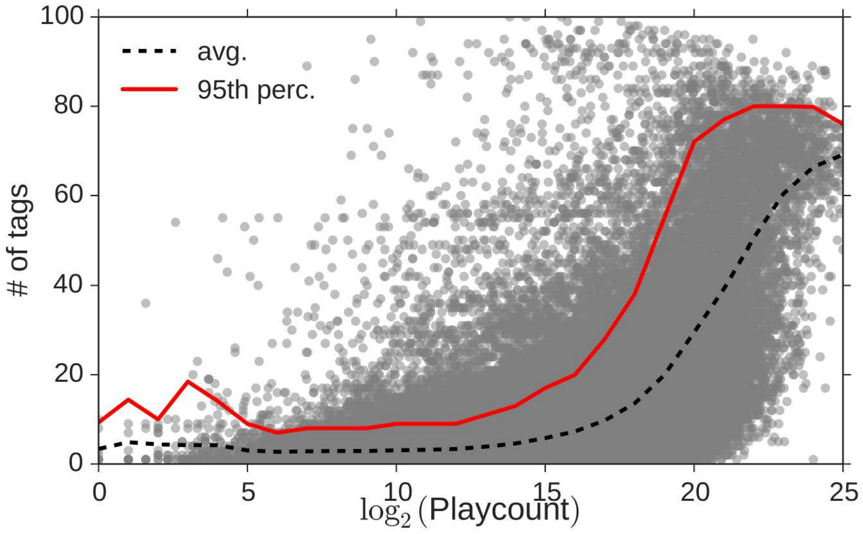

Figure A: Number of tags as a function of the logarithm of the number of Playcounts of an album in the dataset. The black dotted line represents the average number of tags given a certain value of Playcount, while the red continuous line shows the 95th percentile of the distribution of the number of tags.

B Example of Percentage of Tags for a Set of Topics

In Fig. B we show the fraction of the total number of tags belonging to a subset of topics from 1950 to every year until 2015. We here recall that topics are defined as communities in the network of tags (see main text, section Materials and Methods, for details). Here each tag is considered with its multiplicity, i.e., counting a tag as many times as the number of albums it has been associated with. We identify each topic with its most frequent tag appeared until 2015. In total we found 4,340 topics, few of them being very representative while lots of them being minor topics with approximately a thousand tags.

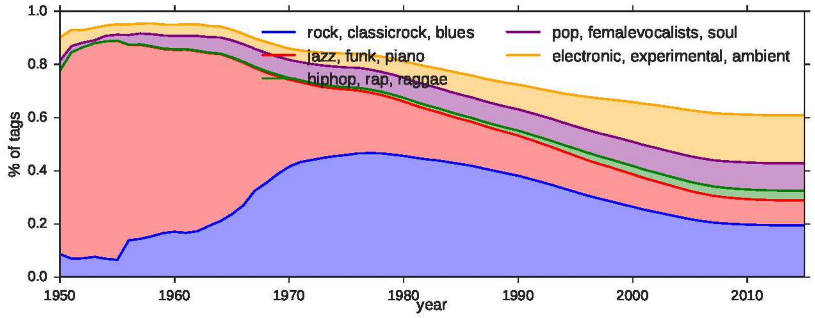

Figure B: Fraction of tags belonging to a set of topics per year. More explicitly, in the y-axis we report each year, for the five most representative topic, the ratio: (# of tags from 1950 until the year)/(# tags in the topic from 1950 until the year). In the legend we show the most frequently used tags for each topic.

C Definitions of the Album Metrics

In the main-text we briefly introduced some metrics that we used to characterize albums as well as their relation and impact on the global musical production. Here we discuss their definitions in detail.

C.1 Topical Entropy

First appeared in [1], this measure was used to classify Twitter Hashtags according to their topic in order to predict their future success. Considering an album a we can compute the fraction of tags belonging to it with a certain topic l. Calling this fraction $f(l)$ and being L_a the set of topics of the tags belonging to a, we can compute the entropy of the distribution of the tags in L_a as $h_a = -\sum_{l \in L_a} f(l) \log f(l)$, that is a measure of how heterogeneously the tags are distributed over the different topics. We further define the normalized entropy, that takes values between 0 and 1, as:

$$E(a) = \frac{h_a}{\log |L_a|}, \tag{1}$$

being $|L_a|$ the number of different topics belonging to a. We will call this metric "Topical Entropy" or just "Entropy" of an album.

C.2 Average Time Span

While the Topical Entropy quantifies the heterogeneity of an album in its topics, we can define a similar metric measuring how heterogeneous is the album over tags belonging to different years. The set of the tags of a, T_a, defines a subgraph of $\mathcal{G}$ which is a directed clique. Considering the edges of this clique E_a, each edge $e \in E_a$ connects two tags with a difference in their creation time $\Delta y(e) = |y_{tag_2} - y_{tag_1}|$. We can define the "Average Time Span" as

$$TS(a) = \frac{\sum_{e \in E_a} \Delta y(e)}{|E_a|}. \tag{2}$$

Note that if all the tags in T_a had been created in the same year, then $TS = 0$. The larger TS the more far in time the tags of a have been created and thus a connects parts of $\mathcal{G}$ generated in different years.

C.3 Average Tag Age

The temporal heterogeneity of an album is not a sufficient information in order to characterize the time frame of its inspirations and styles. In fact, if the album is not heterogeneous and its tags are very related to a specific period of time, then it is not certain that this period of time would be recent as we expect to happen for the albums of a revival band. Hence, we define a metrics aiming at quantifying how far from the release date of the album is the average age of its tags. We call this metrics "Average Tag Age", defined as

$$A(a) = \frac{\sum_{tag \in T_a} (y_a - y_{tag})/(y_a - 1950)}{|T_a|}, \tag{3}$$

where $A = 0$ if all the tags have been created in the release year y_a of the album and it will be larger if they are older.

C.4 Mainstreamness

If an album a has been created in the year y_a, we can consider all the tags of all the albums a' so that $y_{a'} < y_a$ and define the global distribution $F(l)$ of such tags in the space of all the possible topics. This distribution represents the global musical production until the year y_a and hence we can define

$$M(a) = \frac{1}{\sqrt{2}} \|\sqrt{f} - \sqrt{F}\|_2, \tag{4}$$

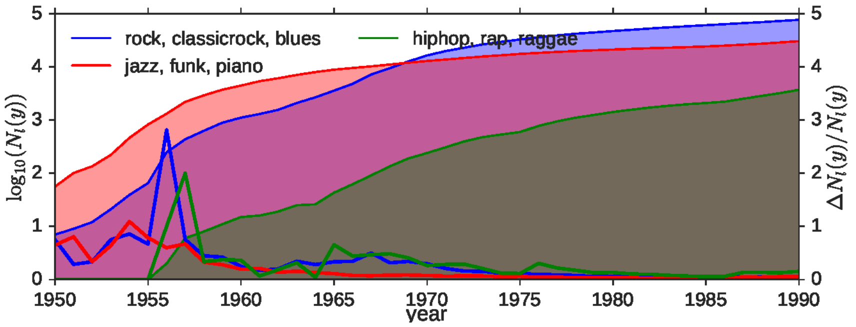

Figure C: Time series of $N_l(y)$ for some topics l (curves with filled area below) and their corresponding logarithmic derivative (thick curves of the same color). In the legend we show some of the most frequently used tags in each topic.

representing how far is the album a from such production. This metrics is basically the Hellinger Distance between the topical distribution of an album and the global distribution $F(l)$. Since $M \in [0,1]$, a value of $M = 1$ indicates that the album has a distribution of tag topics completely different from the global one, while $M = 0$ indicates that the album adheres completely to the current musical style. We call this metric "Mainstreamness" since it gives an indication of how different was the album a from everything that had been produced since its creation year.

C.4.1 Burstiness

Different topics might have a very different history, with blooming of tags associated to them happening in different years. We can define the number of tags (with repetitions) of a given topic l in the year y, $N_l(y)$. Hence, we can define the relative production in that year as

$$\frac{\Delta N_l(y)}{N_l(y)} = \frac{N_l(y+1) - N_l(y)}{N_l(y)}, \tag{5}$$

which is the logarithmic derivative of $N_l(y)$. A strong increase in the production is marked as a high value of this derivative, while a production which is almost steady will have a small value of the derivative (this metrics has already been widely used to measure bursts in on-line popularity, see for instance [2]). Fig. C shows some example of the growth of $N_l(y)$ for some topics and the corresponding logarithmic derivatives. We then define the "Burstiness" B_a of an album a as

$$B(a) = \sum_{l \in L_a} f(l) \frac{\Delta N_l(y)}{N_l(y)}, \tag{6}$$

where y is the release year of the album a. In this way an album having many tags with topic l in a year of large values of $\frac{\Delta N_l(y)}{N_l(y)}$ will have a large B_a. The burstiness measures to which extent an album contributes to the growth of topics that witness an expansion in the year of the album release.

C.5 New Tags

The number of new tags that an album injects in the system when it is created is a simple metrics indicating how innovative the album is. Hence, given the set of tags before the year of creation of the album a, $T(y_a - 1)$, the set of newly introduced tags by a will be $T_a \setminus T(y_a - 1)$. Thus, we can define the fraction of newly introduced tags in the network $\mathcal{G}$ as

$$f_{new}(a) = \frac{|T_a \setminus T(y_a - 1)|}{|T_a|}. \tag{7}$$

Note that more than one album could have introduced the same tag in the same year. Here we will neglect possible effects due to multiple contributions, even though in principle it is possible to weight each tag according to the number of albums sharing it in its year of creation.

C.6 Adjacent Possible Expansion

We here define the adjacent possible metrics $Adj(a)$, measuring how much of the conceptual space has become accessible after the creation of an album a. To this end, given the tag co-occurrence network $\mathcal{G}$, we can define for each year y a set of tags being part of the adjacent possible as those tags which have not been yet created, but all their "precursors" have and hence are just one step away from being created (see the main text for a cartoon). More in particular, a node *tag* is in the adjacent possible in the year y ($tag \in A(y)$) iff *tag* will enter in the network after the year y ($y_{tag} > y$), and all the tags tag' linked to *tag* such that $y_{tag'} < y_{tag}$, have already being created in the year y ($y_{tag'} < y$). We can then identify all those tags that entered in the Adjacent Possible in a certain year y as $\gamma(y) = A(y) \setminus A(y-1)$.

Let us now consider a $tag \in T_a$ of an album a and the set of its neighbours on $\mathcal{G}$, indicated as Γ_{tag}. The set of the non-actualised neighbours of *tag* at time y_a will be $\Omega_{tag}(y_a) = \{tag' \in \Gamma_{tag} : y_a < y_{tag'}\}$. In order to quantify how much space *tag* unlocks, we can define the fraction of non-actualised tags that have entered in the adjacent possible during y_a as:

$$f_{Adj}(tag, y_a) = \frac{|\Omega_{tag}(y_a) \cap \gamma(y_a)|}{|\gamma(y_a)|}. \tag{8}$$

Hence, we define the "Adjacent Possible" metrics for an album a as the average over all the tags in T_a:

$$Adj(a) = \frac{\sum_{tag \in T_a} f_{Adj}(tag, y_a)}{|T_a|}. \tag{9}$$

In this way, $Adj = 1$ indicates that all the tags of an album have contributed to make accessible all its future neighbours, while $Adj = 0$ indicates that either those neighbours where already in the adjacent possible or they did not enter in the adjacent possible during y_a. The Adjacent Possible metrics has been first defined in [3], where it has been used to study the evolution in time of the worldwide movie production.

C.7 Uchronia and Uchronia Entropy

Among the other metrics defined in [3], the "Uchronia" metrics was used to characterize the long-term impact of a movie over the overall movie production. Here we introduce two slight variations of this metrics, one of which exploits the definition of the tag topic.

Considering a tag *tag* created at y_{tag}, we can study the cascade effect of its removal supposing that it will influence all the tags semantically related to it and created afterwards. Starting from an initial set $R = \{tag\}$, we proceed as follows:

- We pick a tag, say tag_1, from R and remove it from the network $\mathcal{G}$ and from R itself.
- For each tag_2 so that (tag_1, tag_2) is a directed edge of $\mathcal{G}$ from tag_1 to tag_2, we compute the quantity:

  $$\alpha(tag_2) = \frac{\sum_{tag' \neq tag_1} w(tag', tag_2)}{\sum_{tag'} w(tag', tag_2)}, \tag{10}$$

  which is the fraction of the weight of the links pointing to tag_2 remaining after the removal of tag_1.
- if $\alpha(tag_2) < \alpha_{thresh}$, where α_{thresh} is a fixed parameter, we put tag_2 in R.
- Iterate from the first point until $R = \emptyset$.

At the end of this procedure some tags might have been removed from the network so that we end up with a reduced version $\mathcal{G}'$ of it, that will have a different distribution F' of the tags in the topics. Note that in the main text we used $\alpha_{thresh} = 1$ so that the removal of just one precursor will lead to the removal of another tag. Considering that a tag can affect only the tags created after it, we define the "Uchronia" metrics as

$$uch(tag) = \frac{|\{tag' : tag' \in \mathcal{G}' \wedge y_{tag'} \geq y_{tag}\}|}{|\{tag' : tag' \in \mathcal{G} \wedge y_{tag'} \geq y_{tag}\}|}, \tag{11}$$

i.e., the fraction of nodes survived after the removal of *tag*.

Another possible quantification of the disruptive effect of the removal of a tag can be the "Uchronia Entropy Variation",

$$h_{uch}(tag) = \frac{h(F) - h(F')}{\log |L|}, \tag{12}$$

which is the difference of the entropies of the distribution of the tags over the topics before and after the removal, where $|L|$ is the number of topics in $\mathcal{G}$. Note that $h_{uch} > 0$ will indicate that the final system is more clustered over a set of topics than the initial one and otherwise $h_{uch} < 0$ will indicate a more uniform distribution and thus a more disordered system.

We can now define the "Uchronia" metric for an album a as:

$$uch(a) = \frac{1}{|T_a|} \sum_{tag \in T_a} uch(tag) \left(1 - \frac{N(tag, y)}{N(tag)}\right), \tag{13}$$

where $N(tag, y)$ is the number of albums sharing that tag until the year y, and $N(tag)$ is the same number in 2015. In this way, the Uchronia metric of a tag will have a larger impact over the Uchronia metrics of early adopters of the tag with respect to the others. Similarly, the Uchronia Entropy Variation of an album a will be defined as:

$$h_{uch}(a) = \frac{1}{|T_a|} \sum_{tag \in T_a} h_{uch}(tag) \left(1 - \frac{N(tag, y_a)}{N(tag)}\right) \tag{14}$$

D Random Forest Predictions on Randomized Data

The significance of the predictions of the Random Forest Algorithm in the main text can be assessed by a comparison with the same predictions performed on a randomized version of the sample. In Fig. 5 of the main text, we have shown that many of the metrics introduced in this work have a strong dependency on time. Similarly, many of the lists used to create the categories used for the classification task exhibit some bias in time (e.g. the Rolling Stone and Grammy Hall of Fame lists have a albums realeased mainly from 1960 to 1980). In order to generate a random sample, taking into account these temporal biases, we divide the sample of the albums in tranches according to their release decade. Hence, whithin each decade we randomly reassing the Playcount values among the albums as well as the belonging to the Rolling Stone, NME, Grammy Hall of Fame and National Recording Registry lists. In this way, we built a random sample preserving the distributions shown in Fig. 5 (dashed lines) of the main-text. We then proceed to built the same 4 categories, *Popular* , *Highly Popular*, *RS/NME* and *GHF/NRR* and use the Random Forest Algorithm to predict the belonging of an album to one of them. We find that:

- The AuROC for the *Popular* and *Highly Popular* categories is respectively 0.52 and 0.53 indicating that the prediction is very close to being random.
- The AuROC for the *RS/NME* is 0.47, again very close to the random classification.
- The AuROC for the *GHF/NRR* is 0.6, which is not as close to random as the previous cases but still is considerably smaller with respect to the non-randomized case 0.81.

These results confirm that the prediction obtain with our metrics and the RFA are significantly different from a randomized case.

E Random Forest Predictions on with a Different Community Detection Algorithm

The computation of some of the metrics introduced in Section C (namely the Mainstreamness, Burstiness, Topical Entropy and Uchronia Entropy metrics), requires the categorization of albums' tags by means of a community detection

algorithm on the co-occurrence network. Throughout the main text, we arbitrarily used the Louvain method [4] and we showed how the metrics defined with this algorithm are predictive of the *Popular*, *Highly Popular*, *RS/NME* and *GHF/NRR* categories. In order to test the robustness of the method, we recomputed all the metrics using the OSLOM community detection algorithm [5] and preformed all the classifications with the Random Forest Algorithm again. The predictive power of our metrics if slightly enhanced by the use of this more recent community detection algorithm:

- The AuROC for the *Popular* category is 0.86 compared to the 0.85 in the main text;
- The AuROC for the *Highly Popular* category is 0.95 (0.92 in the main text);
- The AuROC for the *RS/NME* category is 0.96 (0.89 in the main text);
- The AuROC for the *GHF/NRR* category is 0.77 (0.81 in the main text).

Hence, we can argue that there is not a strong dependence on the community detection algorithm used to perform the categorization of tags. Moreover, in Fig. D we show the rankings of the metrics used in the Random Forest Algorithm according to their relevance in the prediction (the same as in Fig 6 in the main text). In every case we can see that the 3 most relevant metric are the same, besides their order in the ranking which may vary, hence showing a good degree consistence with the previous results with the Louvain method.

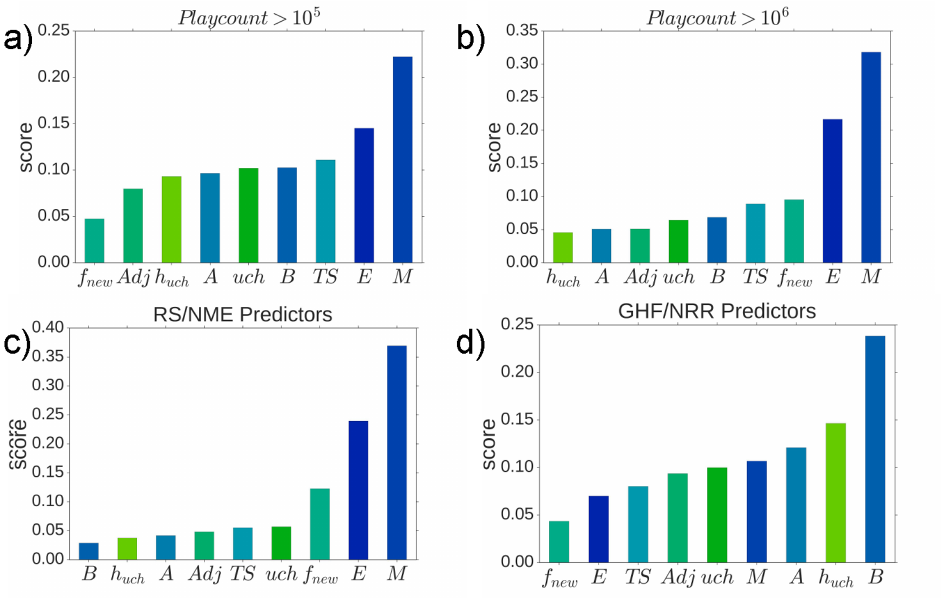

Figure D: Ranked Scores of the Metrics used for the prediction of the 4 categories (a) Popular, (b) Highly Popular, (c) RS/NME, (d) GHF/NRR, where the metrics have been computed using the OSLOM community detection algorithm [5].

F Metrics vs Success

In the main-text we have seen that many metrics are slightly correlated or anti-correlated with the Playcount which is our proxy for success. We here report in Table A the Spearman's Correlation Coefficients for the different metrics. In Figures E, F and G, we show these dependences more in detail, both for the whole set of albums, and separately for the four categories defined in the main-text. Note that the relations of these metrics with respect to the Playcount are diverse so that not a unique general pattern can be derived from these figures. Some metrics, e.g. Mainstreamness (Fig G) seems to have a maximum for a certain value. This might indicate an optimal value of the distance between an

Table A: **Spearman's Correlation Coefficients between the album metrics and the Playcount. All the p-values were found to be orders of magnitude smaller than 0.05.**

Metrics	Spearman's Coeff.
E	0.245
M	-0.366
B_a	0.030
A	-0.063
TS	0.333
f_{new}	0.285
Adj	0.170
uch	0.229
h_{uch}	-0.079
Age	-0.142

artwork and the global artworks production in order to maximize the appreciation of the public, in agreement with [6] in which crowd-sourced tags were used to characterize the production of movies.

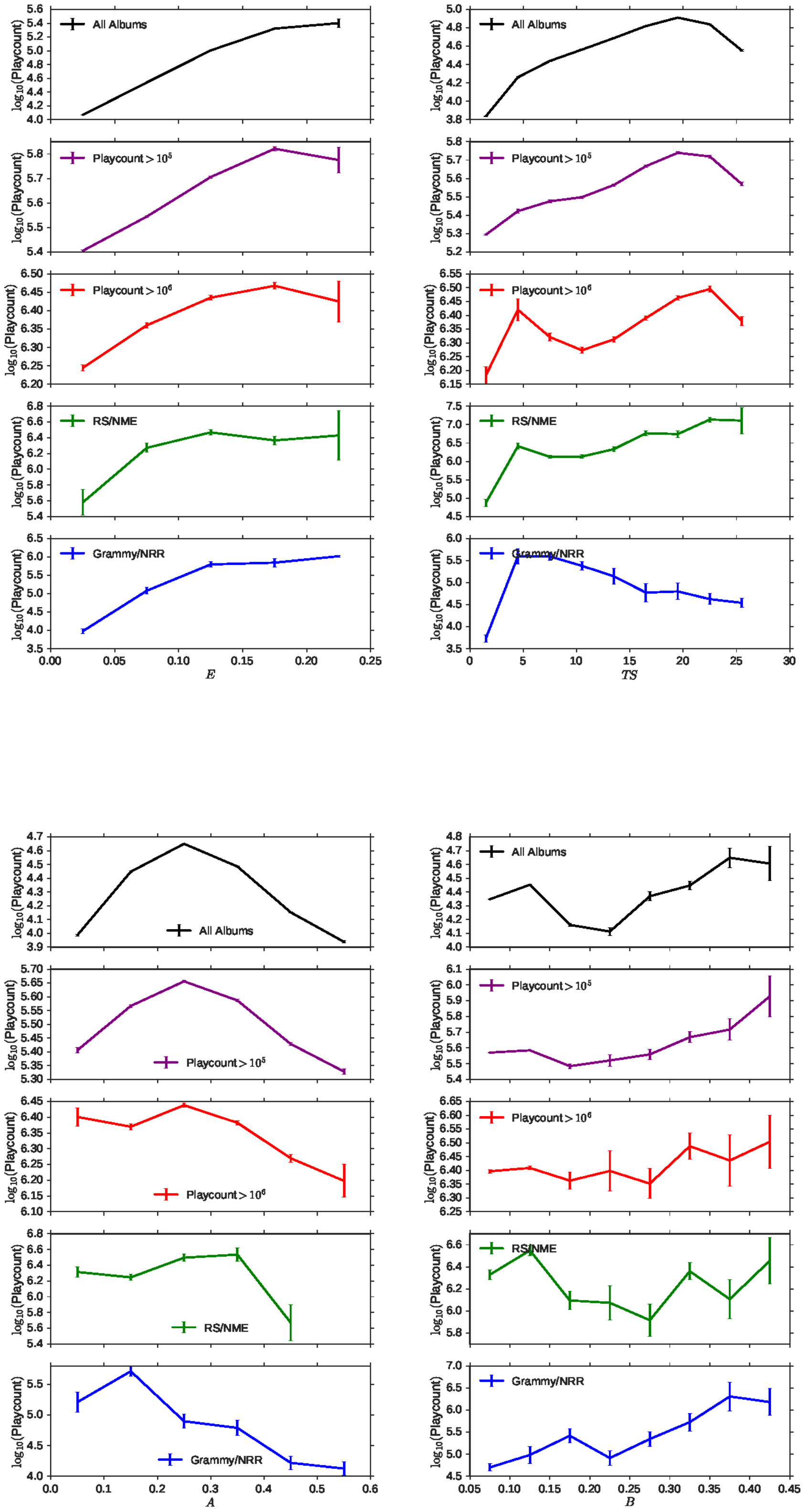

Figure E: Topical Entropy (upper left), Average Time Span (upper right), Average Tag Time (bottom left) and Burstiness (bottom right) as functions of Playcount.

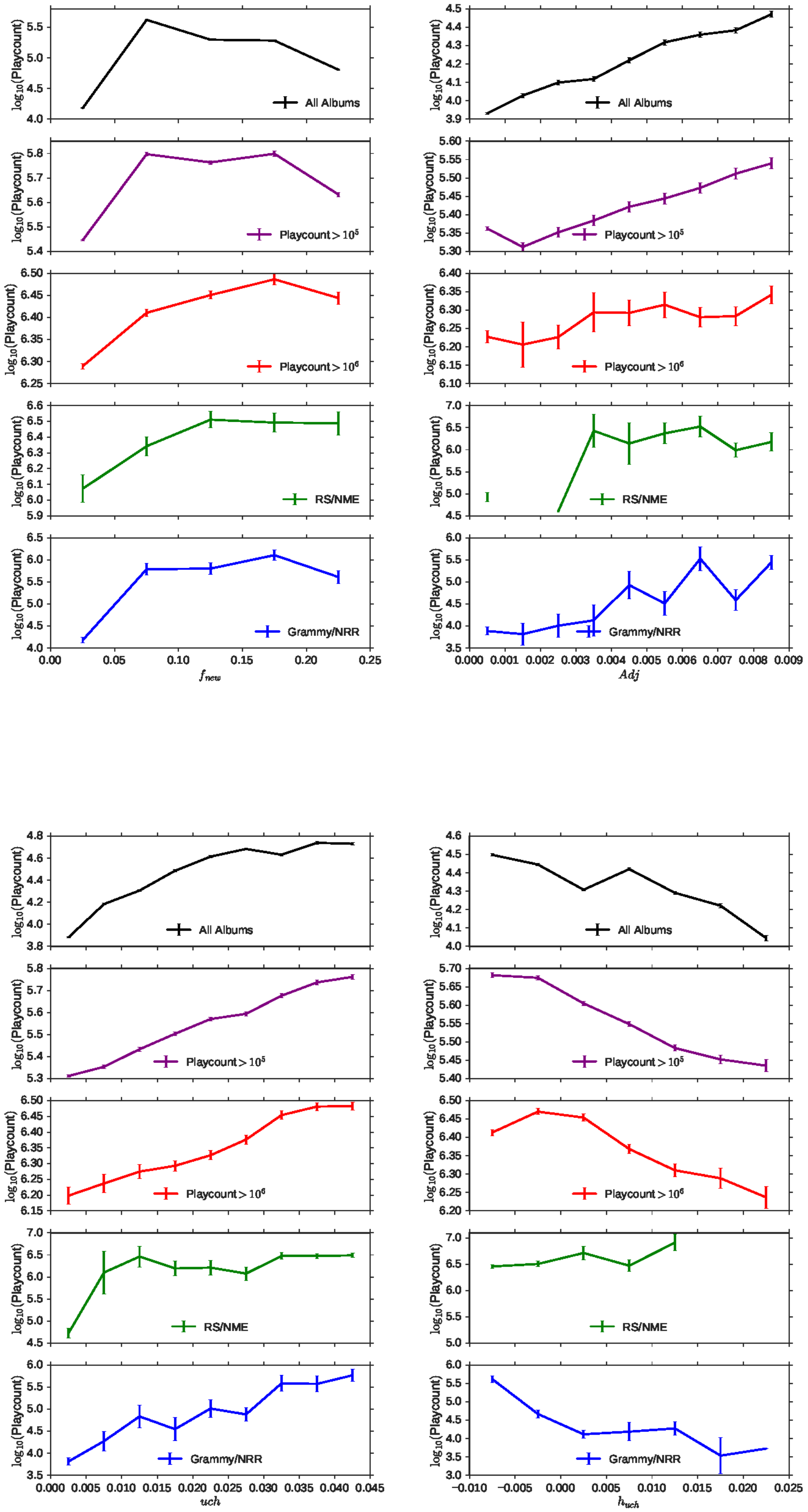

Figure F: Novelty (upper left), Adjacent Possible (upper right), Uchronia (bottom left) and Uchronia Entropy (bottom right) as functions of Playcount.

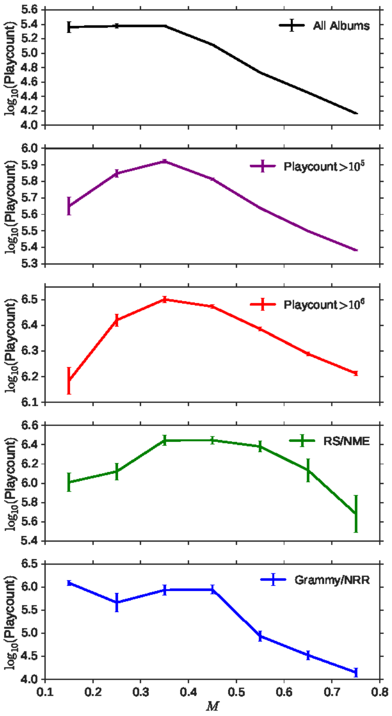

Figure G: Mainstreamness as function of Playcount.

G Adjacent Possible Metrics

The adjacent possible metrics is the only metrics in the main-text that has not found to be relevant for any prediction. Still, its importance might have been covered by some most relevant metrics. In Fig. H, we show the same graphs presented in the main text for the 4 defined categories with the Adjacent Possible metrics (see Figures 7,8,9 and 10 of the main-text). We see that typically there is an increased probability of belonging to one of the categories for values of Adj which are different than 0, indicating that significant or popular albums usually have contributed to the unlocking of some parts of the Adjacent Possible. We speculate that for other categorizations more related with the "avant-gardes" in music, the Adjacent Possible metrics might have a more important role.

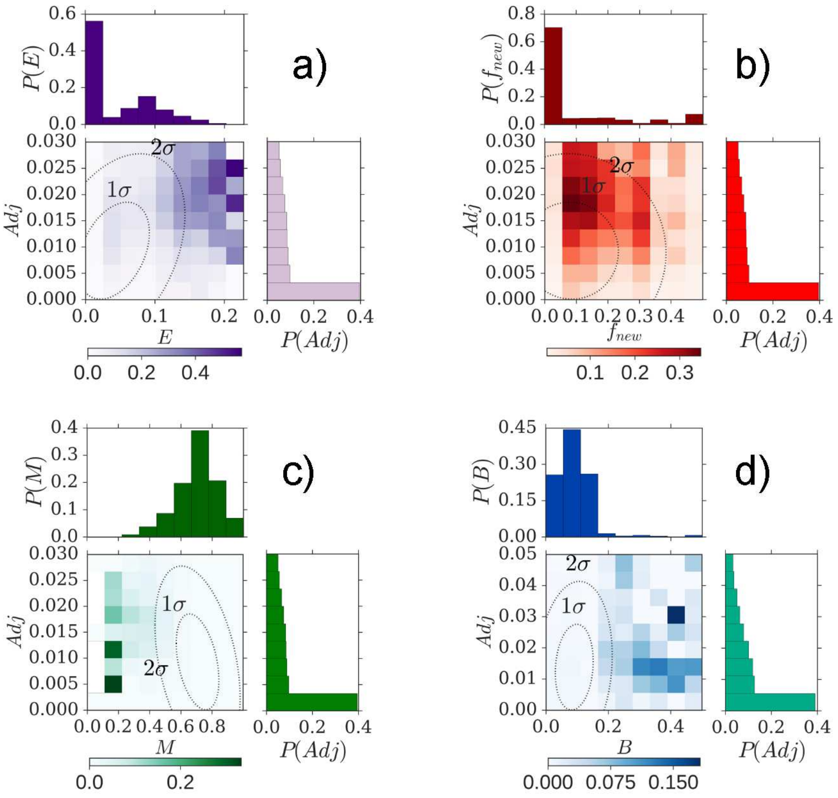

Figure H: Probability of being in the Popular (a), Highly Popular (b), RS/NME (c) and GHF/NRR (d) categories as function of the highly ranked metrics for the RFA prediction and the Adjacent Possible Metrics. Similarly to the figures in the main-text, we show the distribution of the metrics, together with the ellipses of covariance at 1 and 2 standard deviations.

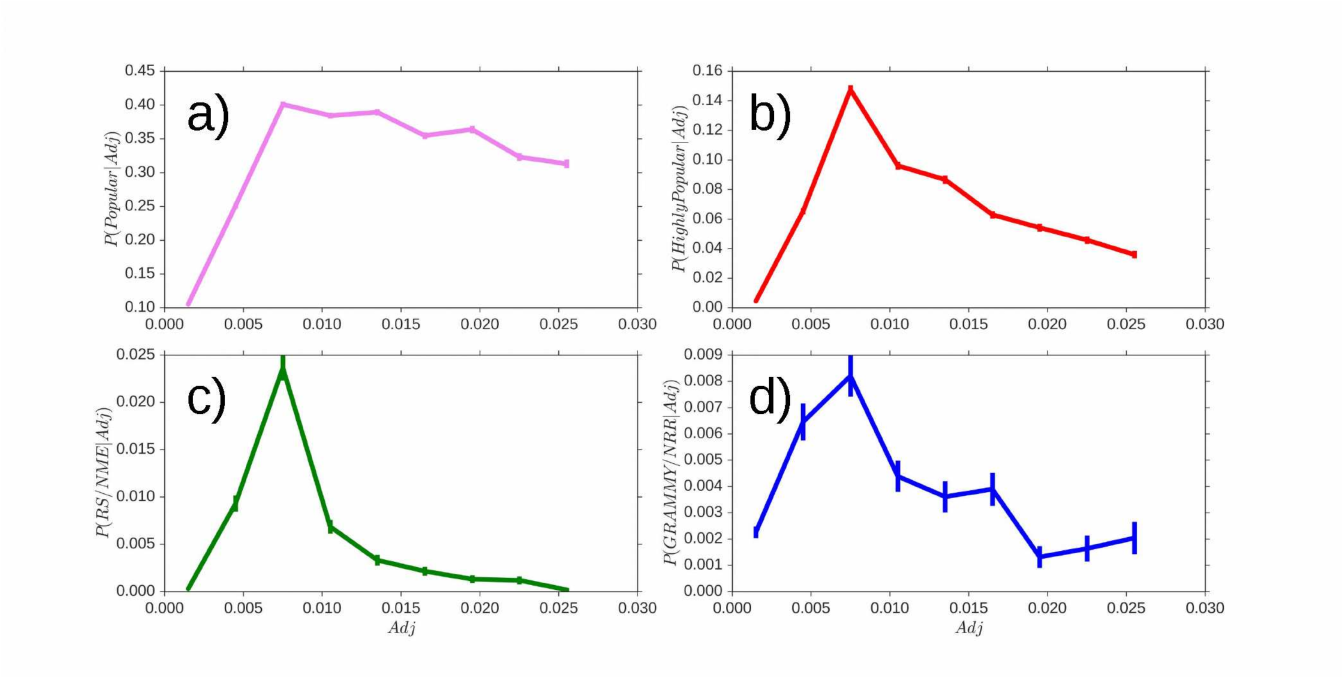

Figure I: Probability of being in the Popular (a), Highly Popular (b), RS/NME (c) and GHF/NRR (d) categories as function of the Adjacent Possible Metrics.

References

[1] Weng, L. & Menczer, F. Topicality and impact in social media: diverse messages, focused messengers. *PloS one* **10**, e0118410 (2015).

[2] Ratkiewicz, J., Fortunato, S., Flammini, A., Menczer, F. & Vespignani, A. Characterizing and modeling the dynamics of online popularity. *Physical review letters* **105**, 158701 (2010).

[3] Gravino, P., Monechi, B., Servedio, V., Tria, F. & Loreto, V. Crossing the horizon: exploring the adjacent possible in a cultural system. In *Proceedings of the Seventh International Conference on Computational Creativity* (2016).

[4] Blondel, V. D., Guillaume, J.-L., Lambiotte, R. & Lefebvre, E. Fast unfolding of communities in large networks. *Journal of statistical mechanics: theory and experiment* **2008**, P10008 (2008).

[5] Lancichinetti, A., Radicchi, F., Ramasco, J. J. & Fortunato, S. Finding statistically significant communities in networks. *PloS one* **6**, e18961 (2011).

[6] Sreenivasan, S. Quantitative analysis of the evolution of novelty in cinema through crowdsourced keywords. *Scientific Reports* **3**, 2758 (2013).
